# Optical coherence tomography and electroretinography in pituitary macroadenomas: a 12-month analysis by age and tumor type

**DOI:** 10.3389/fendo.2025.1694823

**Published:** 2025-12-17

**Authors:** Monika Sarnat-Kucharczyk, Małgorzata A. Janik, Paweł Janik, Beata Kos-Kudła, Dorota Pojda-Wilczek

**Affiliations:** 1 Department of Ophthalmology, Faculty of Medical Sciences in Katowice, Medical University of Silesia, Katowice, Poland; 2 Professor Kornel Gibinski University Clinical Centre, Katowice, Poland; 3 Institute of Biomedical Engineering, Faculty of Science and Technology, University of Silesia in Katowice, Sosnowiec, Poland; 4 Department of Endocrinology and Neuroendocrine Tumours, Department of Pathophysiology and Endocrinology, Medical University of Silesia, Katowice, Poland

**Keywords:** optical coherence tomography, ganglion cell-inner plexiform layer, electroretinography, photopic negative response, W-ratio

## Abstract

**Purpose:**

To evaluate longitudinal changes in optical coherence tomography (OCT) and electroretinography (ERG) parameters over 12 months in patients with pituitary macroadenomas, and to assess variation according to age and tumor type.

**Methods:**

This prospective observational study included 36 patients (72 eyes) with newly diagnosed pituitary macroadenomas. Patients were divided into a treatment group (n=23; including 9 prolactinomas (PRL), and 14 non-functioning adenomas [NFPAs]) and an observation group (n=13; all NFPAs). Both groups were further stratified by age (<60 vs. ≥60 years). Retinal nerve fiber layer (RNFL) and ganglion cell–inner plexiform layer (GCIPL) thickness was measured by OCT, while functional assessment included photopic negative response (PhNR) amplitude and W-ratio from full-field ERG. Measurements were performed at baseline and 12 months. Comparative and correlation analyses evaluated longitudinal, age-, and tumor-related differences.

**Results:**

Mean age did not differ between groups (treatment: 57.4 ± 13.0 years; observation: 54.9 ± 17.2). GCIPL thickness showed no consistent group differences, except temporal thinning in observation patients ≥60 years. RNFL thinning was significant in treated patients ≥60 years (average and inferior/temporal quadrants) and in younger treated patients (<60 years) in the superior and nasal quadrants. Observation patients <60 years showed RNFL loss in average and superior values, whereas no significant RNFL changes occurred in those ≥60 years. PhNR amplitude decreased in treated patients ≥60 years, while W-ratio increased in both treated subgroups and in younger observation patients. Strong structure–function correlations were observed in treated patients ≥60 years, particularly between GCIPL average and PhNR amplitude (R=–0.51) and RNFL superior and PhNR amplitude (R=–0.63).

**Conclusions:**

Structural and functional retinal parameters (GCIPL, RNFL, PhNR) may provide supportive information for monitoring visual pathway involvement in pituitary macroadenomas. Differences between PRL and NFPAs were more evident in treated NFPA patients <60 years, suggesting tumor type and age may influence biomarker sensitivity. The absence of such differences in older patients may reflect biological homogeneity or reduced responsiveness of retinal structures. Age should be considered a potential modifier when interpreting OCT and ERG findings across clinical phenotypes of pituitary macroadenomas.

## 1 Introduction

Anterior pituitary neuroendocrine tumors (PitNETs), formerly known as pituitary adenomas (PAs), are a prevalent subtype of sellar tumors (1). With the increasing life expectancy, physicians are likely to encounter a rising number of PA cases in the aging population in the near future (2, 3).

Prolactinomas are the most common functioning pituitary adenomas, characterized by excessive secretion of prolactin. Clinical manifestations include hypogonadism, galactorrhea, and infertility, with management primarily based on dopamine agonist therapy (4).

In accordance with the WHO 2022 classification, non-functioning pituitary adenomas correspond to non-functioning PitNETs (NF-PitNETs); however, the term NFPA remains widely used in clinical and research contexts and is therefore retained throughout this manuscript for clarity and consistency. NFPAs are relatively common anterior pituitary tumors that arise from hormone-producing neuroendocrine cells of the adenohypophysis but do not cause clinical signs of hormonal hypersecretion. Their clinical impact is primarily related to the mass effect of sellar or parasellar expansion, leading to manifestations such as visual disturbances, headache, cranial nerve dysfunction, and hypopituitarism. In some cases, they are discovered incidentally during neuroimaging performed for unrelated reasons (5).

Previous studies have described several mechanisms underlying visual dysfunction in chiasmal compression, including conduction block, demyelination, ischemic injury, hormonal dysregulation and both retrograde and anterograde degeneration (6, 7).

Axonal damage to retinal ganglion cells (RGCs) in chiasmal compression results from retrograde degeneration, whereby injury propagates from the optic chiasm back toward the cell bodies in the retina (8).

Accumulating evidence indicates that optical coherence tomography (OCT) parameters are highly valuable for diagnosing compressive chiasmopathy and for predicting both the likelihood and extent of visual recovery after decompression surgery (9).

Many studies assess changes in the visual system before and after surgery and attempt to estimate the degree of postoperative visual impairment based on baseline parameters. However, these studies often do not specify tumor subtypes, patient sex, or age group divisions (10–12).

The current trend in the literature emphasizes a tailored approach to treating patients across different age groups. Surgery is clearly indicated for endocrinological, ophthalmological, or neurosurgical reasons, due to the potential space-occupying nature of the tumor, although the treatment goals may vary. In younger patients with pituitary macroadenomas, the focus is on aggressive tumor removal, while in elderly patients, greater attention is given to improving any neurological deficits (with visual impairment being the primary target), addressing hormonal deficiencies, and minimizing the risk of complications (13). When managing elderly patients with pituitary macroadenomas, physicians should consider factors such as life expectancy, comorbidities, neurological deficits, and the risk of tumor recurrence (2).

The objective of this prospective study was to assess longitudinal changes in optical coherence tomography (OCT) and electroretinography (ERG) parameters over a 12-month period in patients with pituitary macroadenomas, and to assess how these changes vary according to age and tumor type.

## 2 Methods

This prospective study was carried out at the Departments of Endocrinology and Ophthalmology of a tertiary care hospital in Poland. Patient recruitment took place over a 1.5-year period, from December 2021 to August 2023, with a follow-up duration of 12 months.

The study adhered to the tenets of the Declaration of Helsinki and received approval from the Silesian Ethics Committee, Poland (approval no. PCN/CBN/0022/KB1/124/21).

### 2.1 Study groups and treatment assignment

Participants were recruited from patients admitted to the Department of Endocrinology with a newly diagnosed pituitary macroadenoma. None had received prior treatment—surgical, pharmacological, or radiotherapeutic—before enrolment in the study.

Patients were classified according to hormonal hypersecretion associated with pituitary tumors: prolactin (PRL)-secreting tumor and non-functioning pituitary adenomas (NFPAs).

Diagnosis of PitNETs was established through a combination of clinical, endocrine, and neuroimaging criteria in accordance with contemporary clinical practice. All patients underwent detailed hormonal evaluation to identify or exclude hypersecreting tumors. Based on these findings, patients were categorized as prolactinomas or clinically non-functioning tumors, with NFPAs corresponding to non-functioning PitNETs (NF-PitNETs) in the WHO 2022 nomenclature.

Patients were qualified for surgical treatment if they presented with significant tumor size, radiological evidence of optic chiasm compression, and/or visual field impairment. In contrast, patients with the absence of chiasmal compression, and preserved visual function were managed conservatively with observation. Hormonal activity of the adenoma was also considered in treatment decisions.

All patients in the treatment group underwent neurosurgical resection. For these cases, the diagnosis of PitNET was confirmed by histopathological examination, including routine immunohistochemical staining for pituitary hormones. Lineage-specific transcription factor profiling (PIT1, TPIT, SF1), although recommended in the WHO 2022 classification, was not yet available in routine pathology practice in Poland during the study period and therefore could not be incorporated into the diagnostic workflow.

Patients in the observation group all had NFPAs managed conservatively and thus did not undergo surgery. For these cases, diagnosis was based on characteristic MRI features of pituitary macroadenoma, lack of hormonal hypersecretion on endocrine testing, and multidisciplinary assessment by endocrinologists, neurosurgeons, neuroradiologists, and ophthalmologists. This diagnostic pathway reflects real-world clinical management of untreated macroadenomas, although it does not permit full molecular subclassification under current WHO 2022 criteria.

The treatment group included patients with pituitary macroadenomas requiring intervention, based on clinical indications such as tumor size and hormone levels. These patients underwent surgical and/or pharmacological treatment.

The observation group consisted of patients with pituitary macroadenomas who did not receive active treatment during the study period and were monitored longitudinally. Although not a typical healthy control group, it served as a reference for assessing the effects of therapy on the visual system.

At study entry, all patients had a pituitary macroadenoma confirmed by magnetic resonance imaging (MRI). Medical history and symptom duration were recorded for each participant. A comprehensive ophthalmic examination was performed at baseline and repeated at 12 months, including best-corrected visual acuity (BCVA), intraocular pressure, refraction, slit-lamp biomicroscopy, dilated stereoscopic fundus examination, posterior segment optical coherence tomography (OCT), and electroretinography.

Independent comparisons were performed between the two groups at baseline and at 12 months, while dependent (within-group) analyses assessed longitudinal changes over the follow-up period.

### 2.2 Inclusion and exclusion criteria

Participants were eligible if aged ≥18 years, had BCVA ≥0.1 (decimal Snellen), and no ocular/systemic pathology other than pituitary macroadenoma. Written informed consent was required.

Exclusion criteria included media opacities (cornea, lens, vitreous), retinal pathology (degeneration, diabetic retinopathy, detachment, trauma, nystagmus), refractive surgery or ocular surgery within 6 months, glaucoma, high myopia (refractive error defined as spherical equivalent refractive error greater than −6.00 diopters) hyperopia (>+6.00 D), psychiatric or systemic disorders limiting examination completion, and pregnancy or lactation.

### 2.3 Best corrected visual acuity

Best-corrected visual acuity (BCVA) was measured using Snellen charts at each visit and converted to decimal notation for analysis. Because Snellen-derived decimal values represent an ordinal scale with discrete stepwise increments, descriptive statistics are presented as medians and interquartile ranges.

### 2.4 Visual field assessment

Visual fields were assessed as part of routine clinical evaluation using Goldmann kinetic perimetry (Campus, Haag-Streit, Switzerland) and static automated perimetry (Octopus 600, Haag-Streit, Switzerland) to further delineate chiasmal defects. As visual field outcomes were collected for clinical characterization rather than predefined analytical endpoints, they were not included in statistical correlation analyses with retinal biomarkers.

### 2.5 Optical coherence tomography

Optical coherence tomography (OCT) was performed using a Zeiss Cirrus 6000 system (Zeiss, Germany). Analysis was carried out with Zeiss OCT software (version 11.5.2.545332), which segments the macular ganglion cell–inner plexiform layer (GCIPL) into six sectors:

1. Superior (S),
2. Nasal Superior (NS),
3. Nasal Inferior (NI)
4. Inferior (I),
5. Temporal Inferior (TI),
6. Temporal Superior (TS).

The retinal nerve fiber layer (RNFL) was divided into four quadrants:

1. Superior (S),
2. Nasal (N),
3. Inferior (I),
4. Temporal (T).

**Figure 1:**
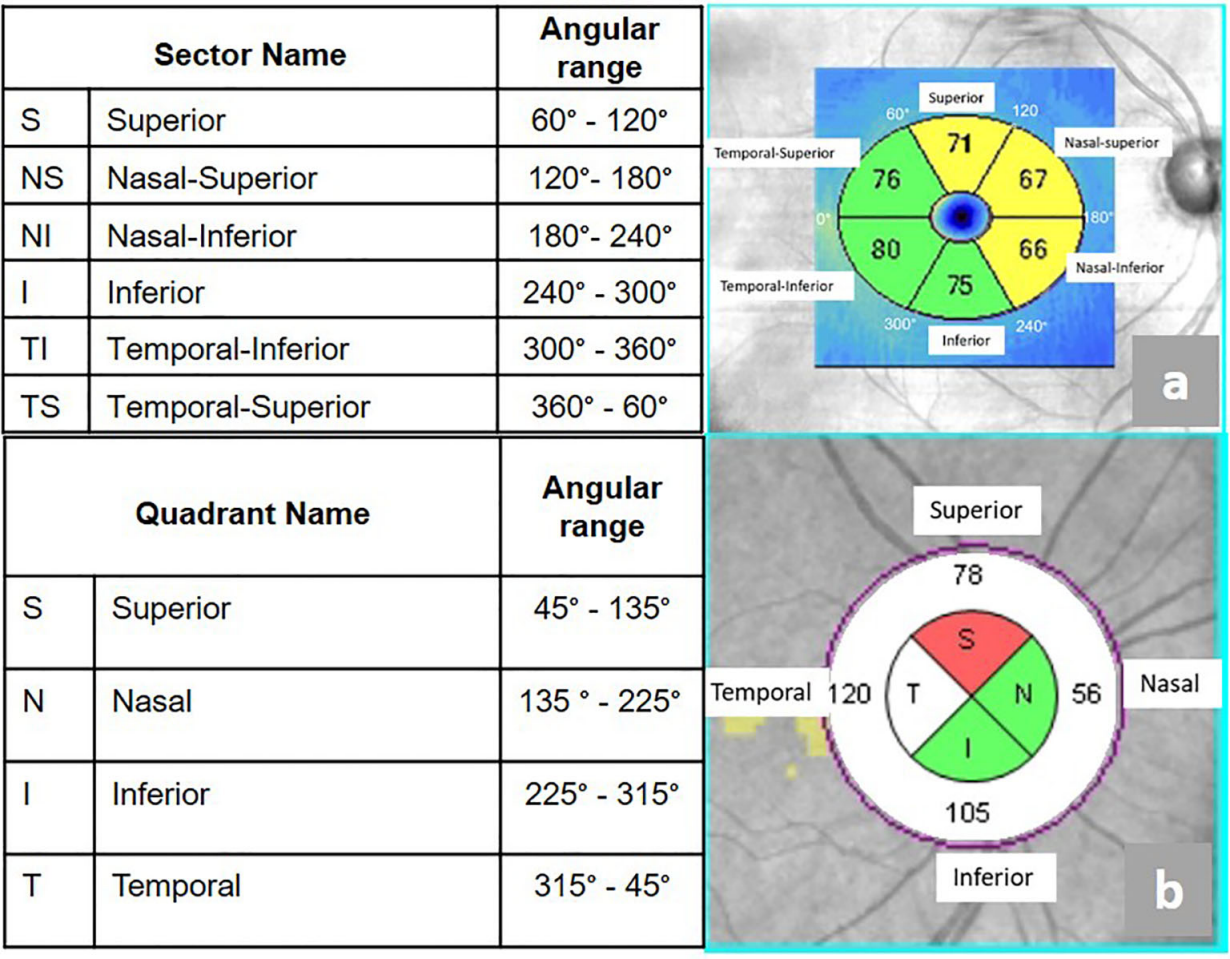
Division of GCIPL and RNFL in optical coherence tomography. **(a)** Macular GCIPL divided into six sectors. **(b)** Peripapillary RNFL divided into four quadrants. Image derived from clinical data of a representative participant who provided written informed consent for publication. Figure prepared by the first author.

The angular ranges and anatomical locations of each GCIPL sector and RNFL quadrant are shown in **Figure 1**.

### 2.6 Photopic negative response in electroretinography

Electroretinography (ERG) was performed using the portable RETeval^®^ system (LKC Technologies, USA) for rapid, non-invasive assessment of retinal function. Photopic ERGs were recorded monocularly with Sensor Strip skin electrodes containing active, reference, and ground elements. The strip was positioned on the lower eyelid, approximately 1 mm below the lid margin, following standard protocols.

**Figure 2:**
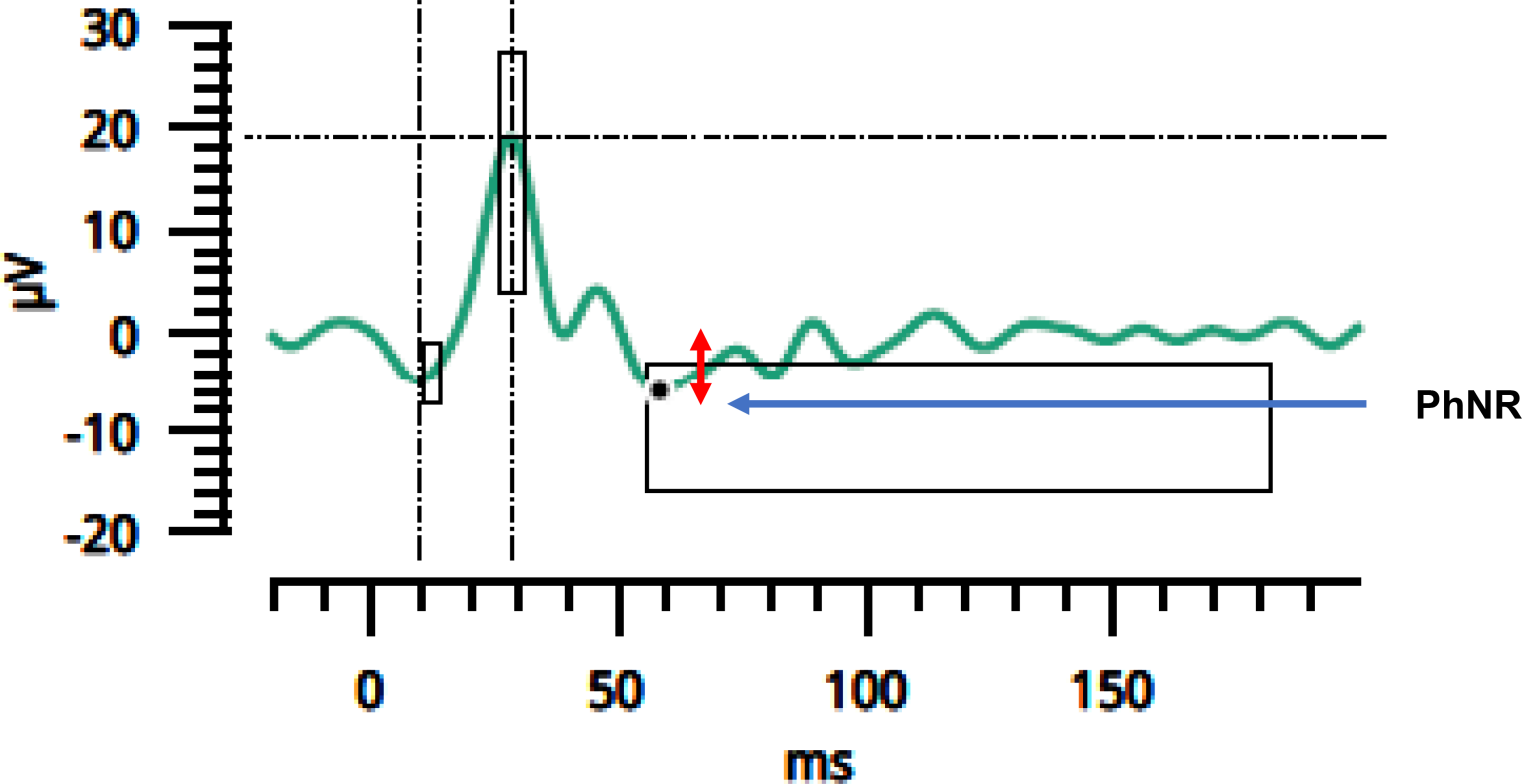
Photopic negative response (PhNR) amplitude. The minimum point of the PhNR, indicated by a black dot, was used for amplitude measurement (red double arrow). μV—microvolts; ms—milliseconds. Image derived from clinical data of a representative participant who provided written informed consent for publication. Figure created by the first author.


The analysis focused on the photopic negative response (PhNR) amplitude and PhNR W-ratio (**Figure 2**), as previously described by Mortlock et al. (14).

$$W-\text{ratio}-\frac{b-p_{\text{min}}}{b-a}$$

Since the PhNR amplitude is conventionally negative, raw (negative) values were used in the statistical analysis.

### 2.7 Statistical analysis

Data were analyzed using *Statistica* 13 (TIBCO Software Inc. (2017). Statistica (data analysis software system), version 13. http://statistica.io.; accessed 27 August 2025). Normality of distribution was assessed with the Shapiro–Wilk test and verified visually using histograms. Homogeneity of variances was evaluated with Levene’s test. Comparisons between two independent groups (treatment vs. observation) were performed using the unpaired t-test or, when assumptions were not met, the Mann–Whitney U test. For repeated measures (baseline vs. 12 months), the paired t-test or Wilcoxon signed-rank test was applied. Associations between variables were examined using Spearman’s rank correlation. A p-value < 0.05 was considered statistically significant.

Patients were divided into two main groups: the treated group and the observational group. Within each group, participants were further stratified according to age (<60 years vs ≥60 years) and tumor type (NFPAs vs PRL). Interaction analyses examined combined effects of these factors.

## 3 Results

### 3.1 Descriptive statistics

The mean age was 57.40 ± 12.99 years in the treatment group and 54.85 ± 17.23 years in the observation group. In the treatment group, males had a mean age of 60.17 ± 11.76 years and females 54.36 ± 13.85 years. In the observation group, mean ages were 54.50 ± 17.90 years for males and 54.91 ± 17.54 years for females.

The distribution of pituitary tumor types in the treatment and observation groups is summarized in **Table 1**, with percentages reported in relation to rows, columns, and the total sample size.

**Table 1:** Distribution of tumor type (Nonfunctioning vs. PRL) in treatment and observation groups by sex.

Group/sex	Nonfunctioning (N/eyes)	PRL (N/eyes)	Nonfunctioning (%)	PRL (%)
Treatment (total)	14/28	9/18	60.9%	39.1%
M	8/16	4/8	66.7%	33.3%
F	6/12	5/10	54.5%	45.5%
Observation (total)	13/26	0	100%	0%
M	2/4	0	100%	0%
F	11/22	0	100%	0%

BCVA remained stable throughout follow-up (median 1.0 at baseline and 12 months; IQR 0.95–1.0), showing a ceiling effect and therefore not included in correlation analyses (**Supplementary Table S1**). When analyzed as an ordinal variable using contingency tables, BCVA did not differ significantly across age strata in either the treatment group (p = 0.8443) or the observation group (p = 0.0805). Only one patient in each group demonstrated reduction in BCVA over the 12-month period. Overall, BCVA remained highly stable across subgroups.

Longitudinal analysis of OCT and ERG parameters is summarized in **Table 2**. GCIPL parameters remained largely stable across subgroups. In contrast, significant RNFL thinning was detected in the treatment group, particularly among patients ≥60 years in the average, inferior, and temporal quadrants, and among those <60 years in the superior and nasal quadrants. In the observation group <60 years, significant changes were observed in the RNFL average and superior quadrants, whereas no significant RNFL alterations were found in the ≥60 years subgroup. A significant reduction in PhNR amplitude was noted in the treatment group ≥60 years, while the PhNR W-ratio increased significantly in both treatment subgroups and in the observation group <60 years.

**Table 2:** Longitudinal analysis of OCT and ERG parameters in the treatment group and the observation group, subdivided by age (<60 vs ≥60 years).

Variable	Group (treatment/ observation)	Age (years)	Baseline (0 mths) Median	Baseline (0 mths) IQR (Q1–Q3)	After 12 mths Median	After 12 mths IQR (Q1–Q3)	P-value (between 0 & 12 mths) – important values
GCIPL Average	Treatment	<60	78	(74-81)	77.5	(73.5-81.5)	0.7368
		≥60	71.5	(68-78)	71	(68-77)	0.5136
	Observation	<60	77	(76-83)	78	(75-84)	0.8068
		≥60	75	(68-79)	73	(67-80)	0.4838
GCIPL Superior	Treatment	<60	78	(73.5-84)	78	(71-84.5)	0.8160
		≥60	71	(62-78)	71	(65-76)	0.4591
	Observation	<60	77.5	(75-84)	79	(76-84)	0.3456
		≥60	75	(71-77)	74	(69-78)	0.5203
GCIPL Nasal -Superior	Treatment	<60	78.5	(71.5-86.5)	78.5	(68-85.5)	0.4209
		≥60	73.5	(65-78)	72	(65-77)	0.1337
	Observation	<60	78	(74-85)	78	(74-86)	0.5541
		≥60	77	(61-82)	76	(62-83)	0.5937
GCIPL Nasal -Inferior	Treatment	<60	77.5	(72-83.5)	76.5	(70.5-82.5)	0.5628
		≥60	70	(63-76)	70.5	(66-76)	0.4144
	Observation	<60	79.5	(71-83)	78	(71-83)	0.1197
		≥60	75	(57-82)	73	(58-80)	0.8523
GCIPL Inferior	Treatment	<60	76	(74-79)	75.5	(71.5-79.5)	0.7628
		≥60	69	(63-75)	69.5	(65-74)	0.6359
	Observation	<60	78	(75-81)	77	(73-83)	0.1355
		≥60	74.5	(67-80)	73	(68-78)	0.2260
GCIPL Temporal -Inferior	Treatment	<60	79	(76.5-81)	79	(75.5-81.5)	0.3655
		≥60	73.5	(68-78)	76	(69-80)	0.5713
	Observation	<60	82	(77-86)	83	(76-86)	0.6949
		≥60	78	(71-82)	79	(71-80)	0.8336
GCIPL Temporal -Superior	Treatment	<60	78.5	(74.5-81)	78	(75-80.5)	0.2046
		≥60	75	(69-78)	74.5	(66-78)	0.7764
	Observation	<60	80	(76-85)	81.5	(74-84)	0.8826
		≥60	76	(72-81)	76	(72-80)	0.0425*
RNFL Average	Treatment	<60	89	(84-96.5)	88.5	(82.5-96)	0.0912
		≥60	84	(80-91)	82	(77-87)	0.0162*
	Observation	<60	86	(81-102)	88.5	(84-104)	0.0302*
		≥60	80	(74-88)	81	(73-86)	0.9397
RNFL Superior	Treatment	<60	115	(102.5-126.5)	109.5	(97.5-121.5)	0.0062*
		≥60	105	(92-110)	104.5	(90-108)	0.7701
	Observation	<60	109.5	(98-130)	110	(103-136)	0.0163*
		≥60	97	(93-106)	99	(88-107)	0.0667
RNFL Nasal	Treatment	<60	76	(67.5-84)	73	(63.5-84.5)	0.0461*
		≥60	69	(66-74)	67.5	(62-72)	0.1008
	Observation	<60	66	(60-74)	70	(62-71)	0.3522
		≥60	62	(55-68)	60	(54-64)	0.6336
RNFL Inferior	Treatment	<60	117	(107-127)	117.5	(106.5-123)	0.4852
		≥60	105	(93-123)	103.5	(83-110)	0.0010*
	Observation	<60	119.5	(111-137)	121.5	(110-132)	0.2900
		≥60	101	(95-118)	109	(95-111)	0.7147
RNFL Temporal	Treatment	<60	56.5	(50.5-62.5)	59	(49-61)	0.9479
		≥60	60	(55-64)	55.5	(52-64)	0.0218*
	Observation	<60	55.5	(52-73)	58	(51-76)	0.0680
		≥60	55	(49-68)	55	(49-67)	0.4255
PhNR Amplitude	Treatment	<60	-4.9	(-6.7 to -4.15)	-6.3	(-7.35 to -5.6)	0.1046
		≥60	-3.9	(-5 to -3)	-5.35	(-7.6 to -3.6)	0.0089*
	Observation	<60	-5.2	(-5.6 to -3.5)	-4.5	(-6 to -4)	0.8099
		≥60	-4.75	(-5.15–3.45)	-4.05	(-5.4–3.35)	0.7642
PhNR W-ratio	Treatment	<60	0.95	(0.885-0.975)	1.109	(1.06-1.175)	0.0006**
		≥60	0.875	(0.84-1)	1.05	(0.91-1.18)	0.0018*
	Observation	<60	0.905	(0.88-0.94)	1.00	(1-1)	0.0006**
		≥60	0.885	(0.835-0.97)	0.95	(0.9-1.075)	0.1477

*significant at p < 0.05.

**significant at p < 0.001.

Mths, months; IQR, interquartile range.

**Figure 3:**
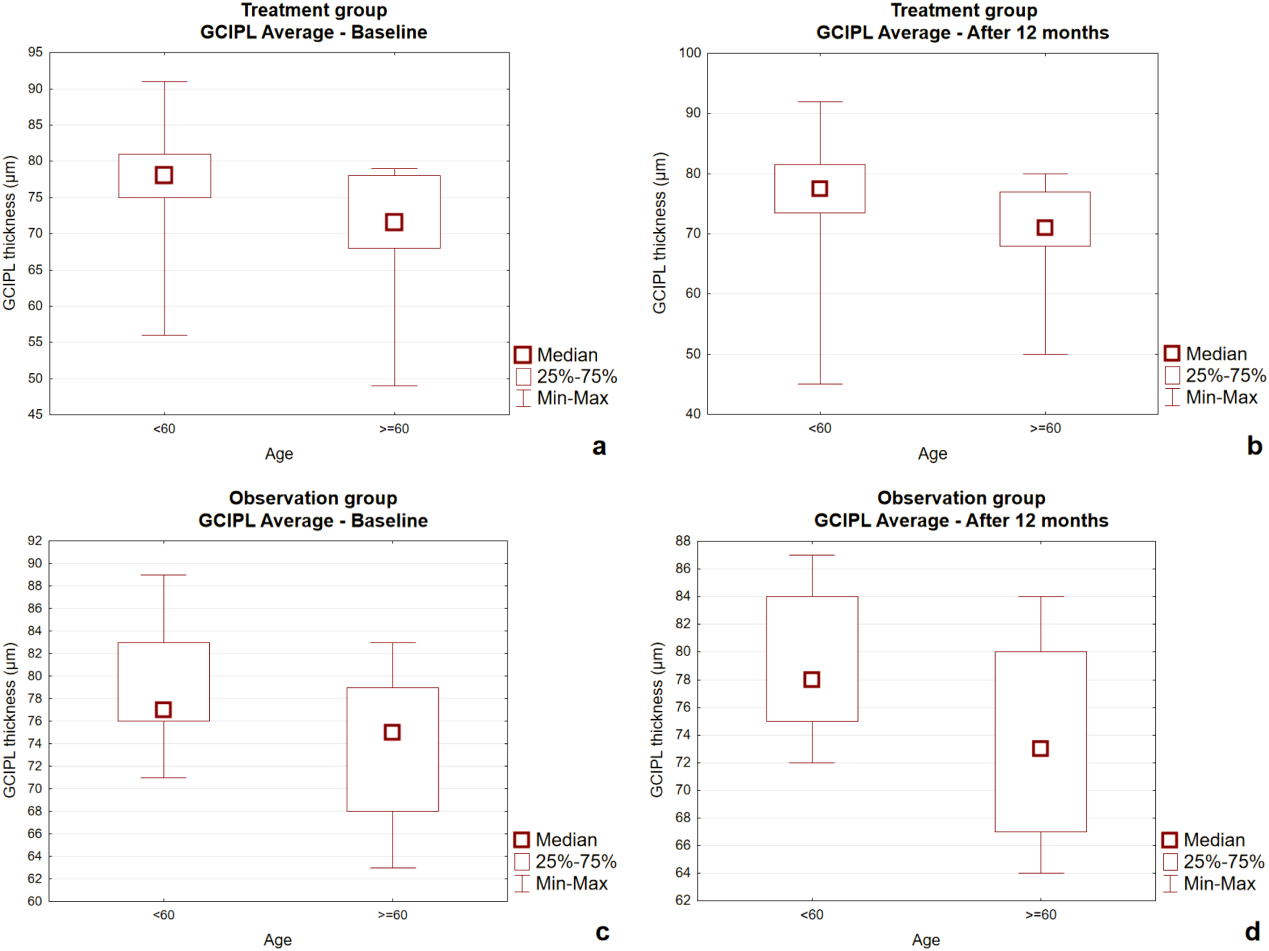
**(a–d)** Box-and-whisker plots comparing the treatment group and the observation group for OCT GCIPL Average at baseline and after 12 months. GCIPL, Ganglion Cell—Inner Plexiform Layer.

**Figure 3** illustrates the distribution of GCIPL average thickness in the treatment and observation groups at baseline and after 12 months, stratified by age (<60 vs. ≥60 years).

At baseline (**Figures 3a, c**), the observation group exhibited generally lower GCIPL values compared with the treatment group, and in both groups’ older patients (≥60 years) showed lower values than younger patients. After 12 months (**Figures 3b, d**), no longitudinal changes in GCIPL average were detected in either the treatment or the observation group, and the overall distribution remained stable across age categories.

**Figure 4:**
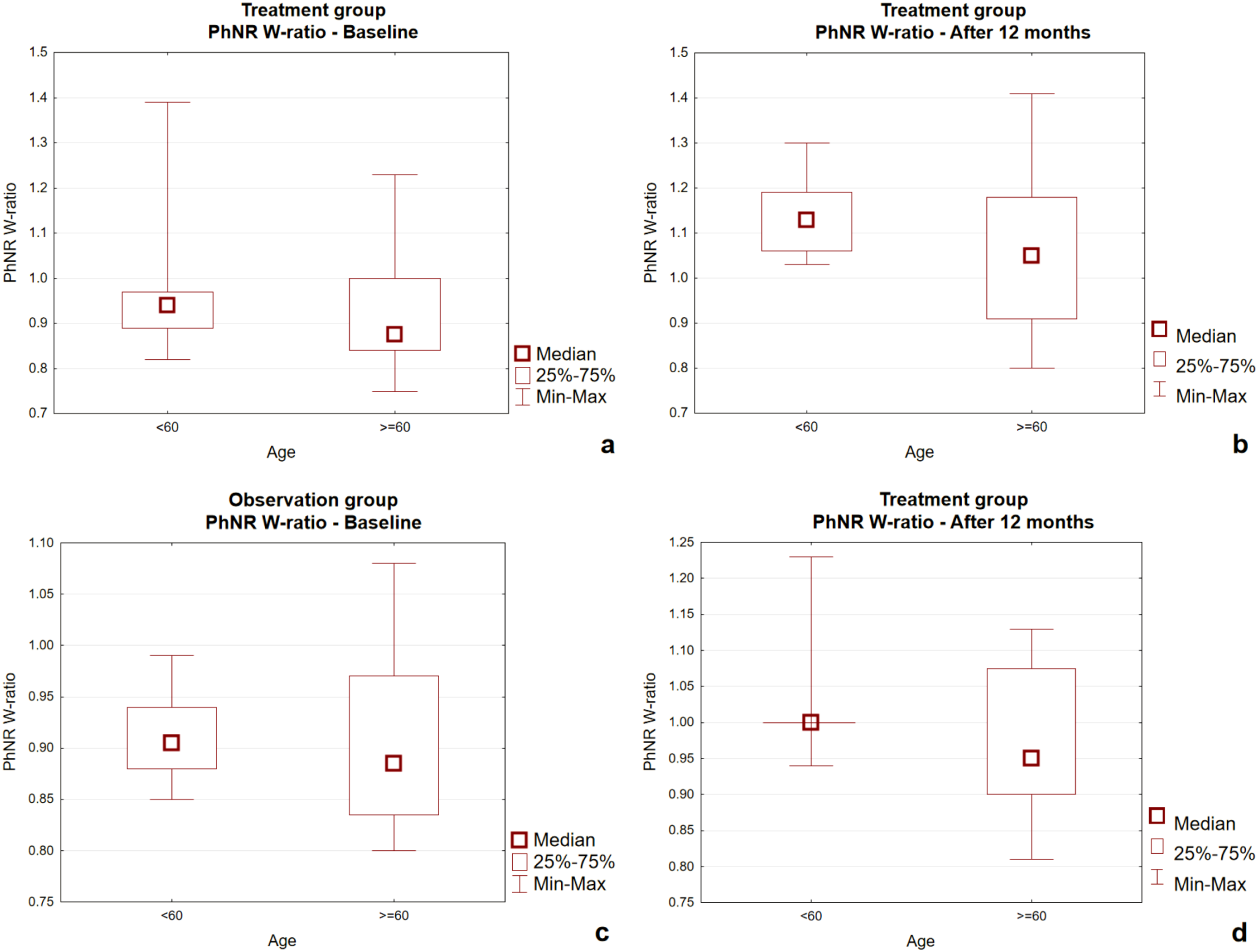
**(a–d)** Box-and-whisker plots comparing the treatment group and the observation group for PhNR W-ratio at baseline and after 12 months. PhNR W-ratio. Photopic Negative Response W-ratio.

**Figure 4** presents box-and-whisker plots of PhNR W-ratio in the treatment and observation groups at baseline and after 12 months, stratified by age (<60 vs. ≥60 years). At baseline (**Figures 4a, c**), W-ratio values were slightly higher in the treatment group compared with the observation group, with younger patients (<60 years) generally showing higher median values than older patients (≥60 years). After 12 months (**Figures 4b, d**), an increase in W-ratio was observed in both age subgroups of the treatment group as well as in the observation group <60 years, while values remained stable in the observation group ≥60 years.

### 3.2 Analytical statistics

The analysis showed no significant age difference between groups (treatment vs. observation, p = 0.6247). The male-to-female ratio differed markedly: 12:11 in the treatment group (near-equal distribution; age difference in this group was statistically insignificant, p = 0.3001) versus 2:11 in the observation group (female predominance; age difference in this group was statistically insignificant, p = 0.9214).

Comparisons between younger (<60 years) and older (≥60 years) patients revealed significant age-related differences in GCIPL measures, particularly for the average, superior, nasal-superior, inferior, and temporal-superior sectors, both at baseline and at 12 months in the treatment group. In the observation group, GCIPL average and superior values also showed significant differences at both time points. RNFL parameters demonstrated age-related effects as well, most notably in the superior and inferior quadrants at 12 months, with additional differences detected for average and nasal sectors in both groups. In contrast, temporal RNFL, PhNR, and W-ratio showed no significant age-related changes in both groups.

Detailed results are provided in **Table 3**.

**Table 3:** Comparison of age groups (<60 vs ≥60 years) in treatment and observation groups.

Variable	Time point (months)	Group					
		Treatment			Observation		
		p	N <60	N ≥60	p	N <60	N ≥60
GCIPL Average	0 mths	0.0022	24	22	0.0243	14	11
	12 mths	0.0110	24	22	0.0398	14	11
GCIPL Superior	0 mths	0.0055	24	22	0.0489	14	10
	12 mths	0.0082	24	22	0.0119	14	11
GCIPL Nasal -Superior	0 mths	0.0088	24	22	0.1884	14	11
	12 mths	0.0227	24	22	0.2494	14	11
GCIPL Nasal -Inferior	0 mths	0.0150	24	22	0.1464	14	11
	12 mths	0.0247	24	22	0.1538	14	11
GCIPL Inferior	0 mths	0.0040	24	22	0.1345	14	10
	12 mths	0.0100	24	22	0.0890**	14	10
GCIPL Temporal -Inferior	0 mths	0.0106	24	22	0.1789	14	11
	12 mths	0.0777**	24	22	0.2163	14	11
GCIPL Temporal -Superior	0 mths	0.0173	24	22	0.2599	14	11
	12 mths	0.0759**	24	22	0.0891**	14	11
RNFL Average	0 mths	0.0473	24	22	0.0348	14	11
	12 mths	0.0077	24	22	0.0108	14	11
RNFL Superior	0 mths	0.0137	24	22	0.0517**	14	11
	12 mths	0.0429	24	22	0.0045	13	11
RNFL Nasal	0 mths	0.0220	24	22	0.2070	14	11
	12 mths	0.0598**	24	22	0.0274	13	11
RNFL Inferior	0 mths	0.1107	24	22	0.0229	14	11
	12 mths	0.0066	24	22	0.0304	14	11
RNFL Temporal	0 mths	0.3110	24	22	0.6612	14	11
	12 mths	0.9913	24	22	0.3229	14	11
PhNR Amplitude	0 mths	0.0086	24	22	0.6066	14	12
	12 mths	0.1592	24	22	0.4824	14	12
W-ratio	0 mths	0.1288	24	22	0.5191	14	12
	12 mths	0.1431	24	22	0.1056	14	12

*statistically significant result with p < 0.05; p **there is a tendency toward statistical significance.

The analysis revealed significant changes in RNFL quadrants between baseline and 12 months across several subgroups. The strongest effects were observed in the treatment group among patients aged ≥60 years, particularly in the RNFL Inferior and Temporal quadrants. Additional significant changes were also noted in patients aged <60 years in both the treatment and observation groups, as presented in **Table 4**.

**Table 4:** Wilcoxon signed-rank test results for RNFL changes from baseline (0m) to 12 months (12m), stratified by group, age, and tumor type.

Group	Subgroup/tumor type	Variable pair	P-value
Treatment	Age <60	RNFL Average 0m & 12m	0.0480
Treatment	Age ≥60	RNFL Average 0m & 12m	0.0178
Observation	Age <60	RNFL Average 0m & 12m	0.0329
Treatment	Age <60	RNFL Superior 0m & 12m	0.0062
Observation	Age <60	RNFL Superior 0m & 12m	0.0192
Treatment	Age ≥60	RNFL Inferior 0m & 12m	0.0010
Treatment	Age ≥60	RNFL Temporal 0m & 12m	0.0218
Treatment	Non-functioning	RNFL Average 0m & 12m	0.0069
Treatment	Non-functioning	RNFL Superior 0m & 12m	0.0461
Treatment	Non-functioning	RNFL Nasal 0m & 12m	0.0339
Treatment	Non-functioning	RNFL Inferior 0m & 12m	0.0361
Treatment	PRL	RNFL Inferior 0m & 12m	0.0280

Comparison of baseline and 12-month results was further stratified in the treatment group by tumor type (non-functioning and PRL). Statistically significant changes were observed mainly in patients with non-functioning tumors, whereas in the PRL subgroup a single significant difference was detected for the RNFL Inferior quadrant (baseline vs. 12 months), also presented in **Table 4**.

Correlation analyses were performed between OCT GCIPL segments and RNFL quadrants and electrophysiological parameters such as PhNR and PhNR W ratio in the treated group, the observation group, and subgroups stratified by age (<60 vs. ≥60 years) and sex (male vs. female) at baseline and after 12 months.

At baseline in the treatment group the highest number of significant correlations was observed in the subgroup of patients aged ≥60 years, which are summarized in **Table 5**. Within this subgroup, however, no correlations were detected when women were analysed separately.

**Table 5:** Correlation between GCIPL/RNFL parameters and PhNR outcomes at baseline and after 12 months in the treatment group (≥60 years) and observation group (total).

Treatment group ≥60 years	Baseline		After 12 months	
Variable pair	R	p	R	p
GCIPL Average & PhNR Amplitude	-0.4774	0.0247	-0.5088	0.0156
GCIPL Average & PhNR W-ratio	0.4581	0.032		
GCIPL Superior & PhNR Amplitude	-0.4932	0.0197		
GCIPL Superior & PhNR W-ratio	0.4230	0.0498		
GCIPL Nasal Superior & PhNR Amplitude	-0.5	0.0178	-0.5472	0.0084
GCIPL Inferior & PhNR Amplitude	-0.4738	0.0259	-0.5178	0.0136
GCIPL Inferior & PhNR W-ratio	0.4413	0.0398		
GCIPL Temporal Inferior & PhNR W-ratio	0.5859	0.0042		
GCIPL Temporal Superior & PhNR Amplitude	-0.4991	0.018		
GCIPL Temporal Superior & PhNR W-ratio	0.4607	0.031		
RNFL Average & PhNR Amplitude	-0.4880	0.0212	-0.6172	0.0022
RNFL Superior & PhNR Amplitude	-0.4939	0.0195	-0.6315	0.0016
RNFL Inferior & PhNR Amplitude	-0.4295	0.046	-0.4801	0.0237
RNFL Inferior & PhNR W-ratio	0.4792	0.024		
Observation group - total	Baseline		After 12 months	
Variable pair	R	p	R	p
GCIPL Average & PhNR W-ratio	0.7300	0.0108		
GCIPL Nasal Superior & PhNR W-ratio	0.8584	0.0007		
GCIPL Nasal Inferior & PhNR Amplitude	-0.6105	0.0461		
GCIPL Nasal Inferior & PhNR W-ratio	0.8838	0.0003		
GCIPL Inferior & PhNR W-ratio	0.7957	0.0059	-0.6646	0.0360
RNFL Average & PhNR W-ratio	0.6073	0.0475		
RNFL Inferior & PhNR W-ratio	0.6515	0.0299		
RNFL Temporal & PhNR Amplitude	-0.6150	0.0440		

In the ≥60-year subgroup of the treatment group, several correlations persisted at 12 months, most notably RNFL superior vs. PhNR amplitude, while other significant associations are summarized in **Table 5**.

In the observation group, the greatest number of significant correlations occurred in the overall cohort at baseline (**Table 5**). Because the group consisted predominantly of women, male patients (n = 2) were not analyzed separately. Notably, correlations between GCIPL nasal inferior vs. PhNR W ratio and GCIPL nasal superior vs. PhNR W ratio were observed only at baseline. However, only the correlation between GCIPL inferior and the PhNR W-ratio persisted after 12 months.

**Figure 5:**
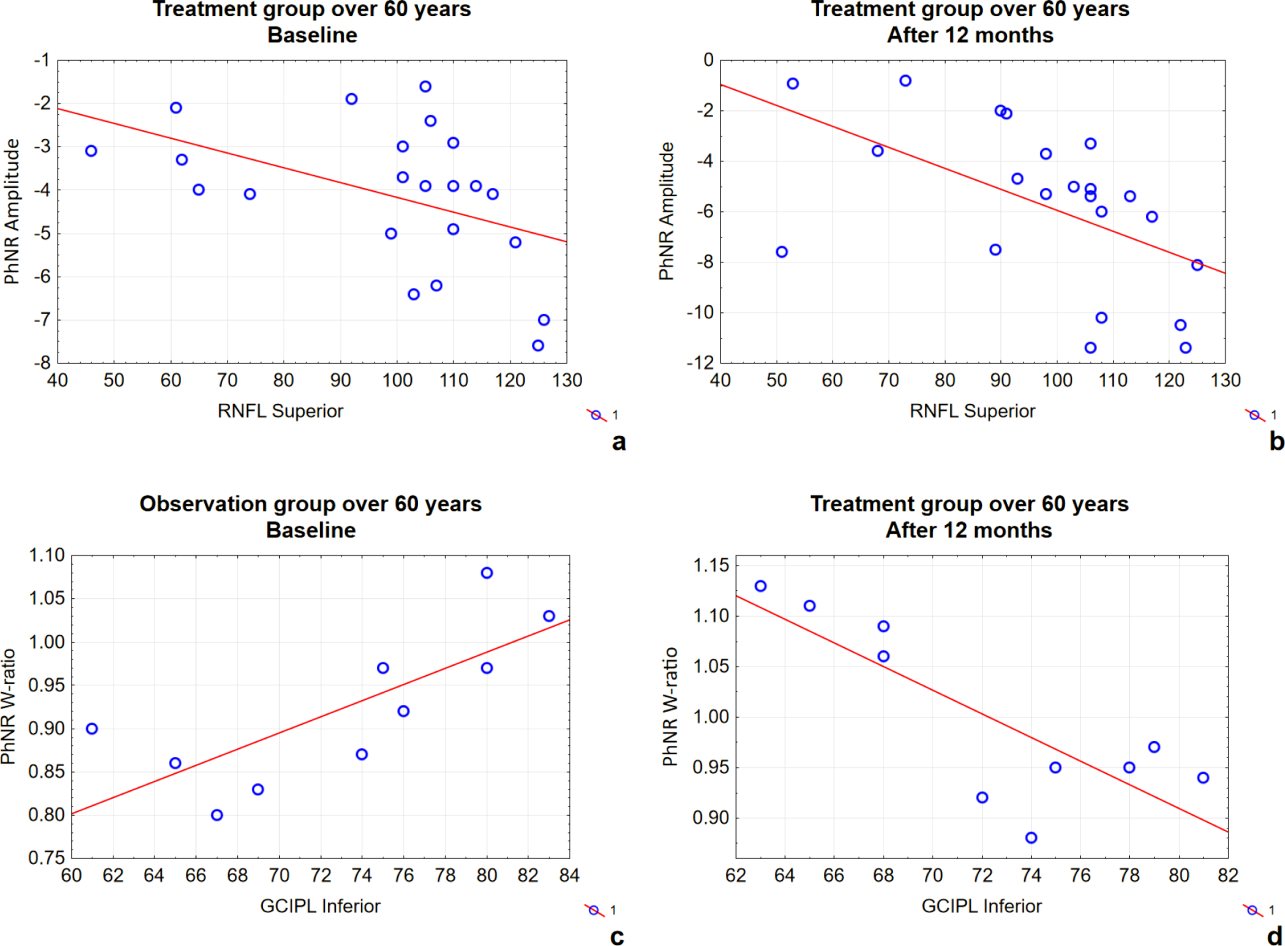
Correlation between structural and functional retinal parameters in subjects over 60 years. **(a)** Treatment group over 60 years at baseline. **(b)** Treatment group over 60 years after 12 months. **(c)** Observation group over 60 years at baseline. **(d)** Treatment group over 60 years after 12 months.

Scatter plots showing the correlation between RNFL Superior thickness and PhNR amplitude in the treatment group over 60 years at baseline (**Figure 5a**) and after 12 months (**Figure 5b**). The correlation between GCIPL Inferior thickness and PhNR W-ratio in the observation group over 60 years at baseline and after 12 months are shown in **Figure 5c** and **Figure 5d**, respectively. Red lines represent linear regression trends.

## 4 Discussion

Our longitudinal data demonstrate that GCIPL parameters remained relatively stable across groups and age categories, suggesting that ganglion cell somas may be less susceptible to short-term change within a 12-month period. In contrast, significant RNFL thinning was detected, particularly in older patients within the treatment group, where the average, inferior, and temporal quadrants were most affected. This finding supports the concept that age-related vulnerability of retinal nerve fiber axons may exacerbate structural changes in the context of pituitary tumors or their treatment (8). In younger patients, significant thinning was also observed, but predominantly in the superior and nasal quadrants, which may reflect regional susceptibility differences rather than generalized axonal loss. Interestingly, in the observation group <60 years, limited but significant changes were noted in the RNFL average and superior quadrants, indicating that progressive alterations can also occur in conservatively managed patients.

Our findings are in line with previous studies showing that RNFL is more sensitive to early changes than GCIPL in patients with optic pathway compression and other optic neuropathies (6, 8, 15–17).

The functional analysis demonstrated a significant increase in PhNR amplitude in the treatment group ≥60 years, indicating improved retinal ganglion cell function (18, 19). The tendency towards a reduction in PhNR amplitude in the untreated observation group <60 years, while not statistically significant, may reflect a direct detrimental effect of pituitary adenoma on retinal ganglion cells and the optic nerves (20). Although the values are negative due to the convention of negative PhNR amplitudes, larger absolute amplitudes in fact indicate better retinal ganglion cell function.

In parallel, the W-ratio increased significantly in both treatment subgroups and in the observation group <60 years. This paradoxical increase may reflect the relative stability of the a-/b-wave compared to PhNR amplitude changes, or compensatory mechanisms affecting the ratio (21). Together, these findings highlight that both structural and functional alterations of the inner retina can be detected over 12 months, particularly in patients undergoing active treatment. In our cohort, the divergence between PhNR amplitude and W-ratio suggests that the ratio does not always mirror true ganglion cell dysfunction (21, 22), but rather reflects the balance between PhNR and a-wave/b-wave components.

Interpretation of the W-ratio therefore remains challenging, and our observations should be regarded as preliminary and hypothesis-generating rather than conclusive evidence of treatment-related functional improvement.

In both groups, patients aged ≥60 years had lower GCIPL values compared with younger participants. This finding is consistent with previous reports showing that inner retinal layers, including the GCIPL, undergo age-related thinning (23–25). Therefore, part of the structural differences observed in our cohort may reflect physiological aging rather than tumor-related effects.

In the treatment group, W-ratio increased over 12 months in both age categories, which may suggest treatment-related changes possibly associated with retinal ganglion cell function.

Similar improvements in PhNR-derived parameters have been reported in interventional studies of glaucoma and optic neuropathies, suggesting that electrophysiological measures can reflect treatment-related functional recovery (23).

Although W-ratio also increased in patients from the observation group, this may be related to various external factors and should be interpreted with caution. The parameter is also influenced by the a- and b-wave components (26, 27), which may partly explain these fluctuations.

Our study demonstrates that GCIPL and RNFL thicknesses are significantly affected by age, with the strongest differences observed in the treatment group.

These findings are in line with previous reports showing progressive thinning of retinal ganglion cell layers with advancing age (28, 29), and support the notion that OCT-derived neuroretinal parameters should be interpreted in the context of patient age (30). The persistence of GCIPL and RNFL differences over 12 months suggests that age-related effects may overlap with disease progression, potentially influencing treatment outcomes (31, 32). By contrast, the absence of significant age effects in temporal RNFL, PhNR, and W-ratio indicates that these parameters may be less sensitive to aging or more strongly influenced by other biological and disease-related factors (33–35). Notably, age-related thinning of the RNFL has been shown to spare the temporal quadrant, a pattern also confirmed in sectoral analyses (36).

PhNR parameters have been shown to remain largely age-independent, with only a weak age correlation observed in certain amplitude components before correction for multiple comparisons (37). Nevertheless, the PhNR is considered to be moderately affected by age overall, and therefore age-matched normative data should be used for accurate interpretation of this measure (38).

Our analysis revealed significant longitudinal changes in RNFL thickness over 12 months, with the strongest effects observed in older patients (≥60 years) in the treatment group, particularly in the inferior and temporal quadrants. These findings are consistent with previous reports indicating that age-related susceptibility of RNFL is most pronounced in the inferior sector, which may reflect its higher vulnerability to both aging and disease-related stressors (36, 39). Interestingly, significant changes were also noted in younger patients and in the observation group, suggesting that RNFL alterations may also represent natural disease progression (40).

Importantly, the observation group comprised patients with non-functioning pituitary macroadenomas who were monitored without surgery rather than healthy controls. In this disease, RNFL changes may be affected by chiasmal compression. Subclinical optic pathway dysfunction may also contribute to the longitudinal changes observed (20, 41). Consequently, differences between treatment and observation groups should be interpreted as contrasts between two clinical phenotypes (treated vs. monitored macroadenomas) rather than treatment vs. health, and residual confounding by tumor characteristics (size, chiasm proximity) cannot be excluded (42, 43). Future studies incorporating a healthy control cohort and detailed structural–functional correlation (OCT/PhNR) are needed to disentangle treatment effects from the natural history of non-functioning macroadenomas.

In accordance with the WHO 2022 classification, definitive subtyping of PitNETs requires immunohistochemistry for pituitary hormones together with lineage-specific transcription factor profiling (PIT1, TPIT and SF1) (3). In our cohort, immunohistochemical profiling for anterior pituitary hormones was performed in all surgically treated patients. However, lineage-specific transcription factor analysis was not available at the time of tissue processing. As a result, we were unable to apply the full 2022 WHO classification framework. This may be relevant, as transcription factor–based classification can reassign tumors previously categorized as “non-functioning” or hormone-lineage–negative based solely on immunostaining. Therefore, the molecular heterogeneity within the NFPA subgroup could be underestimated in this cohort, and future studies incorporating transcription factor profiling may refine tumor subclassification and enhance genotype–phenotype correlations.

Stratification the treatment group by tumor type further demonstrated that patients with non-functioning tumors exhibited multiple quadrant changes, while in the PRL subgroup only a single inferior RNFL change was detected, possibly reflecting differences in tumor biology and neuroretinal involvement (44).

Patients with non-functioning, slowly growing macroadenomas may not notice visual changes for a long time. This delay can result in more severe optic chiasm damage and pronounced alterations in ophthalmic parameters (45). In contrast, prolactin-secreting tumors often cause early hormonal changes, leading to earlier diagnosis before significant chiasmal compression occurs (46).

In this study, we investigated correlations between structural OCT parameters (GCIPL, RNFL) and electrophysiological measures (PhNR, PhNR/W ratio) in patients with pituitary macroadenomas, stratified by treatment status, age, and sex. The most consistent and numerous correlations were observed in the treated subgroup of patients aged ≥60 years, with several relationships persisting after 12 months. In contrast, in the observation group, correlations were most evident at baseline in the overall cohort but were less consistent at follow-up.

Our findings suggest that older patients with treated macroadenomas may show stronger structure–function coupling between OCT and electrophysiological parameters. This may reflect both age-related susceptibility of retinal ganglion cells (47) and the cumulative impact of chiasmal compression (9). The persistence of correlations over time highlights the potential role of OCT and PhNR as complementary biomarkers for monitoring disease progression and treatment outcomes (12).

Previous studies have demonstrated that pituitary adenomas can cause retinal nerve fiber and ganglion cell thinning, which correlates with visual dysfunction (11, 12). Our results extend these observations by showing that specific GCIPL regions, particularly the inferior and superior nasal segments, display robust associations with PhNR outcomes. This aligns with the anatomical vulnerability of crossing fibers at the optic chiasm (48) and their representation in perimacular structures (49).

Interestingly, correlations were largely absent in women within the ≥60-year treatment subgroup. While this finding may be influenced by sample size, sex-related differences in retinal susceptibility (50) or hormonal factors (51) cannot be excluded and merit further exploration. In the observation group, where women predominated, the strong correlations at baseline but weaker persistence after 12 months may reflect more heterogeneous disease trajectories in untreated patients (41).

In the correlation analysis, significant associations were found between structural and functional retinal parameters. In the treated group over 60 years of age, a significant negative correlation was observed between RNFL superior thickness and PhNR amplitude, both at baseline and after 12 months. Importantly, the strength of this correlation increased after one year of follow-up, which may suggest that in this group structural changes within the RNFL are closely linked to an increase in the functional response of retinal ganglion cells. These findings are consistent with previous reports indicating a strong relationship between RNFL thinning and PhNR amplitude in patients with optic nerve neuropathies (52–54). It should be noted that PhNR amplitudes are negative by convention. As raw values were analysed, the correlation with structural parameters appears negative. However, this reflects the mathematical sign rather than a true inverse association, since greater absolute amplitudes actually represent better functional preservation of retinal ganglion cells.

A different pattern was noted in the observation group over 60 years of age, where a positive correlation was initially observed between inferior GCIPL thickness and the PhNR W-ratio, which reversed after 12 months. The disappearance of this association, and even the reversal of its direction, may reflect a different dynamic of changes in the untreated group – possibly related to the natural progression of the disease or compensatory functional mechanisms within the retina (53, 55). Such a reversal of the trend may also suggest that as the disease progresses, structural and functional parameters lose their linear relationship, making it more difficult to predict retinal function solely based on the thickness of cellular layers (56, 57).

### 4.1 Study implications and future directions

The novelty of this study lies in using OCT and ERG as objective, cooperation‐independent biomarkers of visual pathway integrity, offering a complementary alternative to standard subjective functional assessments.

Combined use of these modalities could enhance early detection of subclinical dysfunction and improve monitoring of patients with pituitary macroadenomas, particularly in those at risk for progressive chiasmal damage. To validate these preliminary observations and establish the clinical utility of OCT and PhNR, there is a pressing need for larger, multicenter studies including appropriate control populations.

### 4.2 Limitations and strengths of the study

The relatively small sample size (N = 36), particularly after subdivision into age, sex, and tumor type, and the lack of a healthy control group are important limitations of the study. The small size of the subgroups following stratification further limits the statistical power of these analyses, and findings in these subsets should be interpreted with caution. The lack of a healthy control group further limits the ability to separate age-related retinal changes from those associated with NFPA. Future prospective studies should address this limitation.

Another limitation of our study is the relatively short follow-up period of 12 months. Given the slow and often indolent course of pituitary adenomas, structural and functional changes in the visual pathway may have been underestimated. Longer observation is required to capture the full spectrum of disease-related alterations. Therefore, longer-term prospective monitoring could provide a more complete understanding of the trajectory of retinal structural and functional changes in pituitary adenomas.

A further limitation is that the observation group consisted exclusively of NFPAs, as prospective untreated monitoring is rarely feasible in prolactinomas due to immediate initiation of dopamine agonist therapy. This limits longitudinal comparisons between tumor types.

A notable limitation is the inability to apply full WHO 2022 tumor classification, as transcription factor profiling (PIT1, TPIT, SF1) was not routinely available during the study period.

The main strengths of this study include its prospective, single-center design and the use of a uniform methodology with standardized inclusion criteria, consistent imaging equipment (RNFL/PhNR), and identical examination protocols. Conducting the study within a single center minimized the potential reporting biases and inconsistencies in data collection that are often observed in multicenter trials. Another strength is the relatively long follow-up period of 12 months, which allowed us to assess longitudinal structural changes over time. Furthermore, subgroup analyses stratified by age, sex, and tumor type provided important insights into potential clinical differences that might otherwise remain unnoticed in pooled analyses. To our knowledge, this is one of the few studies to investigate the relationship between PS-OCT, PhNR alterations and tumor type or age stratification in this patient population.

## 5 Conclusions

Our findings suggest that OCT and PhNR have potential as complementary tools for monitoring visual pathway integrity in patients with different clinical phenotypes of pituitary macroadenomas.

Differences observed across age, sex, and treatment groups suggest heterogeneous disease trajectories, underscoring the need for larger longitudinal studies with healthy controls to validate these results.

## Data Availability

The raw data supporting the conclusions of this article will be made available by the authors, without undue reservation.

## Glossary

GCIPL: Ganglion Cell-Inner Plexiform Layer
RNFL: Retinal Nerve Fiber Layer
PhNR: Photopic Negative Response
PhNR W-ratio: Photopic Negative Response W-Ratio
OCT: Optical Coherence Tomography
PRL: Prolactinoma
NFPAs: Non-Functioning Pituitary Tumors
ERG: Electroretinography
RGCs: Retinal Ganglion Cells
S: Superior
TS: Temporal-Superior
TI: Temporal-Inferior
NS: Nasal-Superior
NI: Nasal-Inferior
I: Inferior
T: Temporal
N: Nasal
MRI: Magnetic Resonance Imaging
Mths: Months
IQR: Interquartile Range
ms: Milliseconds
μV: Microvolts
Min-Max: Minium-Maximum
