# Supplementary material for: Optical coherence tomography and electroretinography in pituitary macroadenomas: a 12-month analysis by age and tumor type
Source: Front Endocrinol (Lausanne). 2025 Dec 17;16:1694823. doi: 10.3389/fendo.2025.1694823 (PMC12753404; doi:10.3389/fendo.2025.1694823)
Supplement: Supplementary file 1 [file Table1.docx]

**Supplementary Table 1**. Distribution of BCVA by age in treatment and observation groups at baseline and 12-month follow-up.

| **Group** | **Age** | **Timepoint** | **Median BCVA** | **IQR** |
| --- | --- | --- | --- | --- |
| Treatment | <60 | Baseline | 1.00 | 0.95–1.00 |
|  |  | After 12m | 1.00 | 1.00–1.00 |
|  | ≥60 | Baseline | 1.00 | 0.90–1.00 |
|  |  | After 12m | 1.00 | 1.00–1.00 |
| Observation | <60 | Baseline | 1.00 | 0.95–1.00 |
|  |  | After 12m | 1.00 | 1.00–1.00 |
|  | ≥60 | Baseline | 1.00 | 0.95–1.00 |
|  |  | After 12m | 1.00 | 1.00–1.00 |
